# The global persistence of work from home

**DOI:** 10.1073/pnas.2509892122

**Published:** 2025-07-03

**Authors:** Cevat Giray Aksoy, Jose Maria Barrero, Nicholas Bloom, Steven J. Davis, Mathias Dolls, Pablo Zarate

**Affiliations:** ^a^Office of the Chief Economist, European Bank for Reconstruction and Development and Department of Political Economy, King’s College London, London E14 4BG, United Kingdom; ^b^Business School, Instituto Tecnológico Autónomo de México, Mexico City 10700, Mexico; ^c^Department of Economics, Stanford University, Palo Alto, CA 94305; ^d^Hoover Institution at Stanford University, Palo Alto, CA 94305; ^e^Center for Macroeconomics and Surveys, ifo Institute, Munich 81679, Germany; ^f^Department of Economics, Princeton University, Princeton, NJ 08540

**Keywords:** remote work, post-pandemic work, labor market dynamics, cross-country comparison, working arrangements

## Abstract

Work from home (WFH) surged worldwide during the COVID-19 pandemic, then partially receded as the pandemic subsided. Using our Global Survey of Working Arrangements covering dozens of countries, we find that average WFH rates among college-educated employees stabilized after 2022. The average number of WFH days per week is steady at roughly 1 d per week globally from 2023 through early 2025. Cross-country variation persists: WFH is about twice as common in advanced English-speaking economies as in much of Asia. These results show how the pandemic-driven shift to remote work has persisted and reached a new equilibrium with implications for urban economies, workforce flexibility, and future research on labor markets.

Is widespread working from home (WFH) coming to an end? In the wake of the COVID-19 pandemic, remote work surged to unprecedented levels, becoming a defining feature of modern labor markets. However, as office mandates return and commuting resumes in many cities, questions are mounting about whether the WFH revolution is in retreat—and if so, to what extent and for whom. At the same time, there are persistent cross-country differences in WFH adoption, raising doubts about the global reach and long-term durability of the shift.

Before the pandemic, WFH was relatively uncommon. In 2019, only about 5 to 7% of paid workdays in the United States took place at home; by spring 2020, that share had jumped to nearly 60% during lockdowns (1). Although remote work declined in subsequent years, it remained well above prepandemic levels—accounting for around 28% of paid days in the United States by mid-2023. Globally, the trend is similar, though with significant cross-regional variation: WFH became far more prevalent in English-speaking and Northern European countries but remained limited in much of Asia and Latin America (2, 3). These disparities reflect a mix of institutional, technological, and cultural factors (4). However, until recently, a lack of cross-country data made it difficult to track remote work consistently or assess whether its expansion would continue or plateau.

Understanding where WFH stands today is critical, given its far-reaching implications. A growing body of evidence finds mixed productivity effects—positive in some settings (4–6), negative in others (7, 8). WFH also expands labor supply, especially for women with young children, caregivers, and people with disabilities, who value flexibility and are more likely to work when remote options are available (2, 9, 10). At a macro level, remote work is transforming urban economies—reshaping real estate markets, wage-setting norms, and commuting patterns (11–13). These widespread effects make it essential to track how WFH is evolving—and where it might be headed.

To address these questions, we draw on data from the Global Survey of Working Arrangements (G-SWA)—the only recurring, globally harmonized, stratified survey of remote work. Its most recent wave covers 16,422 full-time, college-educated workers across 40 countries, surveyed between November 2024 and February 2025. The sample spans all major world regions and uses quotas that target the distribution of persons by gender, age, and education by country. These data allow us to examine two key questions: i) Has the decline in remote work continued, or have WFH levels stabilized? and ii) How large are the cross-country differences in remote work adoption?

## Results and Discussion

### Global Stabilization of WFH Rates.

**Fig. 1.**
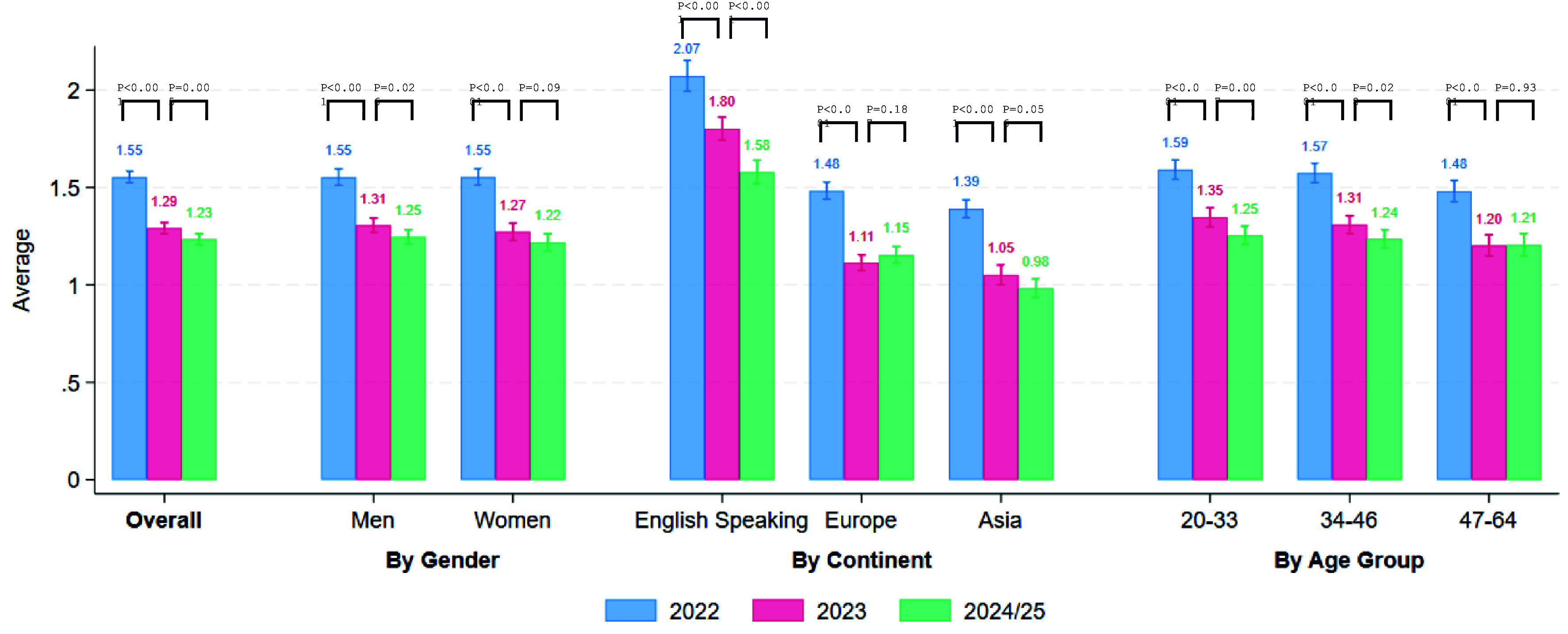
Work from home levels have stabilized since 2023. Note: Responses to: “For each day **last week**, did you **work 6 or more hours**, and if so **where**?”. N = 42,938 college-educated workers in 23 countries surveyed in 2022, 2023, and 2024. Source: G-SWA.

Our central finding is that the overall prevalence of WFH has stabilized since 2023 at the global level. Fig. 1 shows the average number of full paid days worked from home per week in the last three waves of the G-SWA, focusing on the 23 countries surveyed in all three waves. Globally, this average fell from about 1.55 d in 2022 to 1.29 d in 2023 and stands at 1.23 d in late 2024/early 2025 (Wave 4). In percentage terms, this implies that college-educated workers on average do about 25% of workdays from home now. The modest decline from 1.29 to 1.23 d between 2023 and 2024/25 suggests that the postpandemic retreat from remote work has largely bottomed out. Fig. 1 also shows breakdowns by gender, country grouping (discussed further below), and age group. Across all these subgroups, the rate of decline in WFH levels has slowed, with smaller differences between 2023 and 2024/25 than between 2022 and 2023.

This global plateau in WFH echoes patterns observed in other data. For example, office occupancy rates, cell-phone mobility data, and job posting indicators also point toward a stabilization in remote-work levels after 2022 (14). In the United States, a large panel of businesses and workers likewise indicates that WFH settled at just over one-quarter of workdays by 2023.^*^ Our multicountry evidence confirms that a similar stabilization has occurred broadly across the world’s advanced and emerging economies. However, the global average of roughly 1.2 d/wk obscures wide variation in working arrangements across countries as exemplified by the stark differences in WFH levels between English speaking, European and Asian countries in Fig. 1.

### Cross-Country Variation in WFH Adoption.

**Fig. 2.**
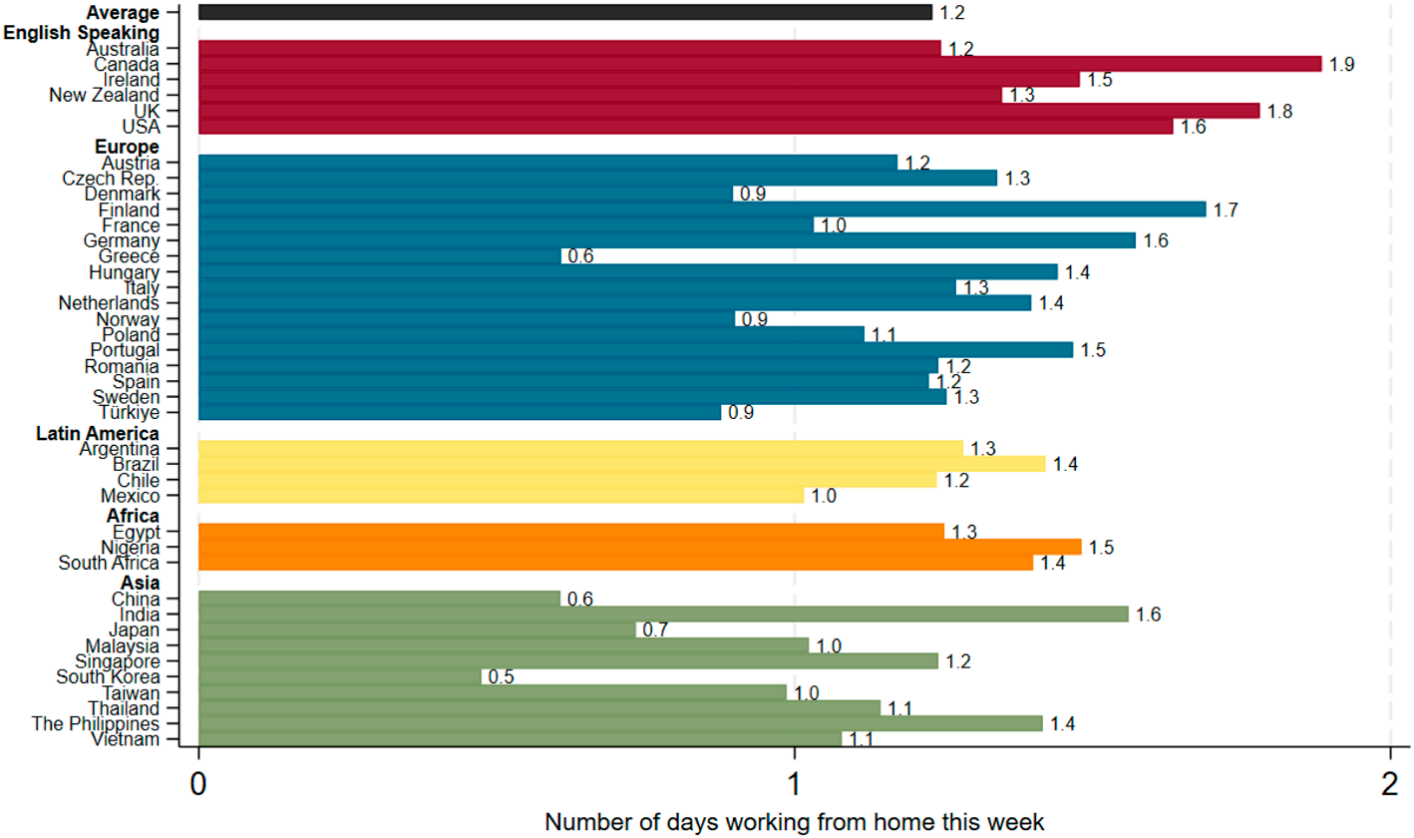
Work from home is more common in North America and Europe and less common in Asia. Note: Responses to: “For each day **last week**, did you **work 6 or more hours**, and if so **where**?”. N = 16,422 college-educated workers in 40 countries surveyed in November 2024 to February 2025. Source: G-SWA.

Rates of work from home continue to vary widely by geography (Fig. 2). For example, college-educated employees in English speaking countries like the United States, Canada, the United Kingdom, and Ireland typically report about 1.5 to 1.9 WFH days per week on average. In contrast, workers in several East Asian countries average well below 1 WFH day per week—especially among that region’s advanced economies. European and Latin American countries fall in between, generally around 1 d per week. These patterns in the 2024–2025 data closely mirror those from our previous 2022 and 2023 survey waves. The rank ordering of countries by WFH levels has remained consistent from year to year. This persistence suggests that structural factors—such as the occupational mix, pandemic experiences, housing markets, and cultural norms—play a significant role in how much work from home happens in each country (15, 16).

## Discussion

Our study provides new evidence that WFH has become a lasting feature of the postpandemic global labor market. Using unique survey data from multiple countries, we find that the share of work performed remotely has been stabilizing since 2023, after declining from its 2020–2022 peak. The world appears to be settling into a new equilibrium, with WFH rates remaining significantly higher than prepandemic levels—even as most work still takes place at employer worksites. Cross-country variation in WFH adoption remains substantial, reflecting structural and cultural factors that warrant further investigation.

## Materials and Methods

### Data and Sample.

We analyze data from the G-SWA Wave 4, conducted between November 2024 and February 2025. The G-SWA is an international survey administered to adult workers via professional survey firms in each country. Wave 4 covers 40 countries, including the United States, Canada, the United Kingdom, dozens of European and Asian economies, as well as a selection of Latin American and African countries. To focus on jobs with WFH potential, the analysis sample targets respondents who are college-educated full-time employees aged 20 to 64. National samples are constructed to be broadly representative of the college-graduate workforce in each country with respect to age, gender, and other demographics (quota sampling is used to ensure balance). The total sample size is 16,422 respondents. In analyses of changes over time (Fig. 1), we restrict to the 23 countries that were surveyed in all three comparison waves (2022, 2023, and 2024/25) to form a balanced panel; results are similar when using all available countries per wave.

Ethics approval was granted by the Chair of the NBER Institutional Review Board (IRB Ref#24_128), and informed consent was obtained from all participants.

## Supplementary Material

Appendix 01 (PDF)

## Data Availability

Survey data will be deposited in Harvard Dataverse (17).

## Footnotes

^*^Monthly time series data on WFH levels from the Survey of Working Arrangements and Attitudes (SWAA) show that WFH levels in the US have stabilized since 2023 (1, 12). Moreover, from around mid-2022, employer plans for WFH 1 y ahead have moved in lockstep with actual WFH rates, providing further evidence of stabilization and long-term persistence in WFH levels (18, 19).

## References

[1] J. M. Barrero, N. Bloom, S. J. Davis, The evolution of work from home. J. Econ. Perspect. **37**, 3–24 (2023).

[2] C. G. Aksoy , Working from home around the world Brook. Pap. Econ. Act. **2022**, 281–360 (2022), 10.1353/eca.2022.a901274.

[3] C. G. Aksoy , “Working from home around the globe: 2023 Report” (EconPol Policy Brief, vol. 53, 2023).

[4] P. Choudhury, B. Z. Larson, C. Foroughi, Work-from-anywhere: The productivity effects of geographic flexibility. Strategic Manage. J. **42**, 655–683 (2021).

[5] N. Bloom, J. Liang, J. Roberts, Z. J. Ying, Does working from home work? Evidence from a Chinese experiment. Q. J. Econ. **130**, 165–218 (2015).

[6] C. G. Aksoy, N. Bloom, S. J. Davis, V. Marino, C. Ozguzel, “Remote work, employee mix, and performance” (NBER Working Paper 33851, 2025).

[7] M. Gibbs, F. Mengel, C. Siemroth, Work from home & productivity: Evidence from personnel & analytics data on IT professionals. J. Polit. Econ. Microecon. **1**, 101–138 (2022).

[8] N. Emanuel, E. Harrington, Working remotely? Selection, treatment, and the market for remote work. Am. Econ. J. Appl. Econ. **16**, 528–559 (2024).

[9] A. Mas, A. Pallais, Valuing alternative work arrangements. Am. Econ. Rev. **107**, 3722–3759 (2017).

[10] N. Bloom, G. Dahl, D.-O. Roth, “Work from home and disability employment” (NBER Working Paper 32943, 2024).

[11] A. Gupta, V. Mittal, S. Van Nieuwerburgh, “Work from home and the office real estate apocalypse” (NBER Working Paper 30526, 2022).

[12] J. M. Barrero, N. Bloom, S. J. Davis, “Why working from home will stick” (NBER Working Paper 28731, 2021).

[13] M. J. Delventhal, E. Kwon, A. Parkhomenko, Work from home and urban structure. Built Environ. **49**, 503–524 (2023).

[14] S. R. Buckman, J. M. Barrero, N. Bloom, S. J. Davis, L. Pinkus, “Measuring work-from-home: Evidence from the American Time Use Survey” (NBER Working Paper 30873, 2023).

[15] J. V. Alipour, O. Falck, S. Schüller, Germany’s capacity to work from home. Eur. Econ. Rev. **151**, 104354 (2023).

[16] P. Zarate , “Why does working from home vary across countries and people?” (NBER Working Paper 32374, 2024).

[17] C. G. Aksoy, Replication data for “The global persistence of work from home.” Harvard Dataverse. 10.7910/DVN/MKS1EI. Deposited 18 June 2025.

[18] J. M. Barrero, N. Bloom, S. Buckman, S. J. Davis, SWAA January 2025 Updates. https://wfhresearch.com/wp-content/uploads/2025/01/WFHResearch_updates_January2025.pdf. Accessed 30 May 2025.

[19] N. Bloom , US executives predict work from home is here to stay. https://siepr.stanford.edu/publications/policy-brief/us-executives-predict-work-home-here-stay. Accessed 30 May 2025.

