# Supplementary material for: The global persistence of work from home
Source: Proc Natl Acad Sci U S A. 2025 Jul 3;122(27):e2509892122. doi: 10.1073/pnas.2509892122 (PMC12260529; doi:10.1073/pnas.2509892122)
Supplement: Supplementary file 1 — Appendix 01 (PDF) [file pnas.2509892122.sapp.pdf]

Appendix for

The Global Persistence of Work from Home

Cevat Giray Aksoy,[1] Jose Maria Barrero,[2] Nicholas Bloom,[3]
Steven J. Davis,[4] Mathias Dolls[5] and Pablo Zarate[6]

[1] European Bank for Reconstruction and Development and King's College London cevat.aksoy@kcl.ac.uk, [2] Instituto Tecnológico Autónomo de México jose.barrero@itam.mx, [3] Stanford University nbloom@stanford.edu, [4] Hoover Institution at Stanford University StevenD5@Stanford.edu, [5] ifo Institute dolls@ifo.de, and [6] Princeton University pzarate@princeton.edu

Supporting Information

The fourth wave of the G-SWA has been fielded in 40 countries from November 2024 to February 2025. The survey includes two equally sized subsamples in each country. The first subsample consists of adults aged 20 to 64, while the second is restricted to full-time working adults aged 20 to 64 who have completed at least secondary education. In France, Germany, Italy, the UK and the US, total sample sizes amount to more than 2,500 respondents, respectively. In all other countries, total samples consist of roughly 1,000 responses.[1] In this report, we restrict attention to full-time workers, aged 20-64, with completed tertiary education, coming from both subsamples.

In addition to basic questions on demographics, employment status, earnings, industry, occupation, marital status and living arrangements, the survey asks about current, planned and desired WFH levels, and more (see survey instrument below). We design the G-SWA instrument, adapting questions from the U.S. SWAA developed by Barrero et al. (2021). We enlist professionals to translate our original English-language questionnaire into the major languages of each country. To ensure high-quality translations, we also enlist an independent third party with knowledge of the survey to review the translations and revise as needed.

To field the G-SWA, we contract with Bilendi (a professional survey firm), which implements the survey directly and in cooperation with its external partners. The survey effort taps pre-recruited panels of people who previously expressed a willingness to take part in research.[2] Recruitment into these panels happens via partner affiliate networks, multiple advertising channels (including Facebook, Google Adwords, and other websites), address databases, and referrals. New recruits are added to the panel on a regular basis. When it is time to field a survey, Bilendi or its partner issues email messages that invite panel members to participate. The message contains information about compensation and estimated completion time but not about the survey topic. Clicking on the link in the invitation message takes the recipient to the online questionnaire. Respondents who complete the survey receive cash, vouchers or award points, which they can also donate.[3]

Before our analysis of the G-SWA data, we drop "speeders," defined as respondents in the bottom 5% of the completion-time distribution for each country. Additionally, we remove those who fail any of three attention check questions, removing another 15% of respondees.[4] After these drops, our analysis sample contains 16,422 observations across the 40 countries in Wave 4. Our samples are broadly representative by age, gender, and education for the group of full-time workers in each country.[5] Completion rates (in %), defined as the number of respondents completing survey wave 4 relative to those who started the survey or dropped out at any point, average 78%, implying that 22% of respondents did not finish the survey. We kept the survey instrument (provided below) as short as possible to minimize attrition. Importantly, additional analyses show that completion rates are not statistically significantly correlated with WFH levels, suggesting that cross-country differences in WFH levels are not driven by differential attrition.

[1] The sample size in India and Nigeria is somewhat smaller and amounts to 875 respondents.
[2] Bilendi and its external partners do not engage in "river sampling," whereby people are invited to take a survey while engaging in another online activity. Relative to river sampling, the use of pre-recruited panels affords greater control over sample composition and selection.
[3] We do not contact respondents ourselves, do not collect personally identifiable information, and have no way to re-contact them.
[4] The questions are: "What is 3+4?", "In how many big cities with more than 500.000 inhabitants have you lived? Irrespective of the truth, please insert the number 33 in order to continue with the survey", and a pair of questions about "Age" at the start of the survey and "Year of birth" at the end.
[5] Respondents take the survey on a computer, smart-phone, iPad or like device, so we miss persons who don't use such devices.

G-SWA – Wave 4 (November 2024 – February 2025)

Target population:

Sample 1: Full-time working age adults (i.e. 20 to 64 years old) who have completed at least secondary education

Quotas: age, gender, education (share secondary/tertiary education)

Sample 2: Adults (20 to 64), irrespective of work status

Quotas: age, gender (50/50), education (primary/secondary/tertiary)

Geography:

Argentina, Australia, Austria, Brazil, Canada, Chile, China, Czechia, Denmark, Egypt, Finland, France, Germany, Greece, Hungary, India, Ireland, Italy, Japan, Malaysia, Mexico, Netherlands, New Zealand, Nigeria, Norway, Poland, Portugal, Romania, Singapore, South Africa, South Korea, Spain, Sweden, Taiwan, Thailand, The Philippines, Türkiye, UK, USA, Vietnam

Legend

Blue = coding instructions

Green = reader notes

Red = termination logic

++++BLOCK 1:

1. How old are you? _______

   [TERMINATE IF YOUNGER THAN 20 or OLDER THAN 64]

2. Which of the following describes your work status?

   [SAMPLE 1 ONLY: TERMINATE IF ANSWER is b, c, d, e, f, g, h, i or j]

   a) Work full-time (30+ hours per week)

   b) Work part-time (up to 29 hours per week)

   c) Apprenticeship, Internship

   d) School

   e) Student

   f) Re-training

   g) Currently unemployed

   h) Pensioner/retired, formerly in full-time work

   i) Not working (housewife/house husband)

   j) Maternity leave, Parental leave, Sabbatical

3. What is the highest level of education you have completed?

[SAMPLE 1 ONLY: TERMINATE IF ANSWER IS a, i.e., IF RESPONDENT DID NOT COMPLETE A SECONDARY SCHOOL EDUCATION]

[Country specific education levels]

Multiple choice | Required | Vertical | Single-select

a) Less than high-school diploma
b) High-school diploma or GED certificate
c) 1 to 3-years of college
d) 4-year college degree
e) Masters or Professional Degree
f) PhD

[Q4 logic: This is an attention check question that serves as an additional screening question. Respondents who do not enter "7" are screened out.]

4. What is 3 + 4?

Number | Required | Min: 0 | Max: 1000000

[TERMINATE IF ANSWER IS NOT "7"]

5. What is your sex?

a) Female

b) Male

c) Other or prefer not to say

6. Approximately how much did you **earn by working in 2023, on a before-tax basis**?

(please report the total **annual** gross income received from wages in main and side jobs)

[Country specific income bands]

a) Less than $5,000

b) $5,000 to $10,000
c) $10,000 to $19,999
d) $20,000 to $29,999
e) $30,000 to $39,999
f) $40,000 to $49,999
g) $50,000 to $59,999
h) $60,000 to $69,999
i) $70,000 to $79,999
j) $80,000 to $99,999
k) $100,000 to $124,999
l) $125,000 to $149,999
m) $150,000 to $199,999

n) $200,000 to $499,999
o) $500,000+

++BLOCK 2:

1. **Last week** what was your work status?

   Multiple choice | Required | Vertical | Single-select | Randomize whether the answer option order is a-b-c-d-e or e-d-c-b-a

   a) Working <u>for pay</u>, whether on business premises or working from home
   b) Employed and paid, but not working
   c) Unemployed, looking for work
   d) Unemployed, awaiting recall to my old job
   e) Not working, and not looking for work

2. For each day **last week**, did you **<u>work 6 or more hours</u>**, and if so **<u>where</u>**?

Matrix | Required | Group by: Row | Single-select | Randomize columns 2-4

Day of the week	Did not work 6 or more hours	Worked **<u>from home</u>**	Worked at **<u>employer or client site</u>**
Monday			
Tuesday			
Wednesday			
Thursday			
Friday			
Saturday			
Sunday			
	Did not work 6 or more hours	Worked **<u>from home</u>**	Worked at **<u>employer or client site</u>**

Set days_worked to Q2 choices by row where value is any of "Worked from home", "Worked at employer or client site"

[Q3 logic: This is an attention check question which aims at boosting attention. Respondents who fail the attention check will receive an error message and have to complete the correct number '33' in order to be able to continue with the survey.]

3. In how many big cities with more than 500.000 inhabitants have you lived? Please note that **this question only serves the purpose to check your attention**. Irrespective of the truth, please insert the number 33 in order to continue with the survey.

   Number | Required | Min: 0 | Max: 100

____

[error message if entered number not '33': In this question we have asked you to insert the number 33. Please read all questions and instructions carefully. Otherwise, your answers cannot be taken into account.**]**

4. Do you currently live with a partner or other adults? (Please check all that apply)

   Multiple choice | Required | Vertical | Multi-select

   a) No
   b) Yes, with a spouse or domestic partner
   c) Yes, with one or more adult children or other relatives
   d) Yes, with roommates

Male or female

show block if Block 1 Q5 selected choice is any of "Female", "Male"

Female fertility

show block if Block 1 Q5 selected choice is "Female"

5. Have you ***given birth*** to any children in ***2015 or later?***

   Q Q_female_birthed | Multiple choice | Required | Vertical | Single-select | Answer option randomization

   a) Yes

   b) No

[Q6 logic: Show if Block 2 Q5 selected choice is "Yes"]

6. How many children have you ***given birth to in 2015 or later?***

   Q_female_children | Multiple choice | Required | Vertical | Single-select

   a) 1 [TAG: 1]

   b) 2 [TAG: 2]

   c) 3 [TAG: 3]

   d) 4 [TAG: 4]

   e) 5 [TAG: 5]

   f) 6 [TAG: 6]

Male fertility

show block if Block 1 Q5 selected choice is "Male"

7. Are you the biological father of any children ***born in 2015 or later?***

Q_male_fathered |Multiple choice | Required | Vertical | Single-select | Answer option randomization

a) Yes

b) No

[Q8 logic: Show if Block 2 Q7 selected choice is "Yes"]

8. How many children ***born in 2015 or later*** are you the biological father of?

Q_male_children | Multiple choice | Required | Vertical | Single-select

a) 1 [TAG: 1]

b) 2 [TAG: 2]

c) 3 [TAG: 3]

d) 4 [TAG: 4]

e) 5 [TAG: 5]

f) 6 [TAG: 6]

Had children since 2015

Show if birthed or fathered any kids since 2015 (Answered Q5 “Yes” OR Q7 “Yes”)

Set num_children_parented to number of children birthed/fathered from Q_female_children OR Q_male_children

[Q9 logic: Show if birthed/fathered 1 child (see responses Q6/Q8)]

9. In what year was that child born?

Combination | Required

Child 1	**Number input**<br>Min: 2015<br>Max: 2024

[Q10 logic: Show if birthed/fathered more than 1 child (see responses Q6/Q8)]

10. In what years were those children born?

Combination | Required	
Child 1<br>Child 2<br>Child 3<br>Child 4<br>Child 5<br>Child 6	**Number input**<br>Min: 2015<br>Max: 2024

11. How many biological children have you had in your life? ___

    Number | Required | Min: 0 | Max: 20

12. Are you currently expecting a baby?

Multiple choice | Required | Vertical | Single-select | Answer option randomization

a) Yes

b) No

[Q13 logic: Show if Q11 is 0]

13. Do you plan to have your own (biological) children someday?

Multiple choice | Required | Vertical | Single-select | Randomize the order of answer options a and b

a) Yes

b) No

c) Don't know

[Q14 logic: Show if Q11 is >0]

14. Do you plan to have another (biological) child someday?

Multiple choice | Required | Vertical | Single-select | Randomize the order of answer options a and b

a) Yes

b) No

c) Don’t know

[Q15 logic: Show if Q13 is “Yes”]

15. How many biological children do you plan to have in total?

Number | Required | Min: 1 | Max: 20

____

[Q16 logic: Show if Q14 is “Yes”]

16. How many more biological children do you plan to have in total?

Number | Required | Min: 1 | Max: 20

____

[Q17 logic: Show if Q13 is “Yes”]

17. At what age ***at the latest*** would you plan to have your first child?

Number | Required | Min: 20 | Max: 100

____

[Q18 logic: Show if Q14 is “Yes”]

18. At what age ***at the latest*** would you plan to have another child?

Number | Required | Min: 20 | Max: 100

____

19. Are there children under 18 living in your household?

Multiple choice | Required | Vertical | Single-select

a) No

b) Yes, 1 child

c) Yes, 2 children

d) Yes, 3 children

e) Yes, 4 or more children

[Q20 logic: Show if Q4 selected choices includes "Yes, with a spouse or domestic partner"]

20. What is your ***spouse or domestic partner's <u>current</u> working status?***

    Multiple choice | Required | Vertical | Single-select | Randomize whether the answer option order is a-b-c-d-e-f or e-f-d-a-b-c

    a) Working from home 1 to 2 days per week

    b) Working from home 3 to 4 days per week

    c) Working from home 5+ days per week

    d) Working all days on business premises

    e) Not working, <u>full time student</u>

    f) Not working, other

[Q21 logic: Show if Q4 selected choices includes "Yes, with a spouse or domestic partner"]

21. What is the highest level of education your partner has completed?

    Multiple choice | Required | Vertical | Single-select

    a) Less than high-school diploma
    b) High-school diploma or GED certificate
    c) 1 to 3-years of college
    d) 4-year college degree
    e) Masters or Professional Degree
    f) PhD

[Q22 logic: Show if Q1 (Block 2) selected choice is any of "Working for pay, whether on business premises or working from home", "Still employed and paid, but not working"]

22. Which of the following best describes your current employment situation?

    Multiple choice | Required | Vertical | Single-select | Randomize whether the answer option order is a-b-c-d or d-c-b-a

    a) I am a **wage and salary employee**, and my <u>main job</u> accounts for most of my earnings

    b) I am a **wage and salary employee** who also earns a lot of <u>extra income from side jobs</u>

    c) I am **self-employed** and run my own business

    d) I earn most of my income as an **independent contractor, freelancer, or gig worker**

23. ***Since 2020*** have you at any point worked primarily from home?

    (For example, because you wanted to, due to lockdowns, or because it was unsafe or otherwise not possible to work on business premises)

Multiple choice | Required | Vertical | Single-select | Answer option randomization

Auto-fill this to "Yes" if Q2 in Block 2 yields at least 1 "Worked from home" day

a) Yes

b) No

24. ***Looking one year ahead,*** how often would you ***like to*** have paid workdays at home?

Multiple choice | Required | Vertical | Single-select | Randomize whether the answer option order is a-b-c-d-e-f-g or reversed order

a) Never

b) About once or twice per month

c) 1 day per week

d) 2 days per week

e) 3 days per week

f) 4 days per week

g) 5+ days per week

25. In what industry is your ***current*** job? If not currently working, then please report the industry of your ***most recent*** job.

Multiple choice | Required | Vertical | Single-select

a) Agriculture, Forestry, Fishing, or Hunting

b) Arts, Entertainment or Recreation

c) Banking, Finance, or Insurance

d) Construction

e) Education

f) Health Care and Social Assistance

g) Hospitality and Food Services

h) Information Services, including Publishing or Media

i) Manufacturing

j) Mining, Quarrying or Oil and Gas Extraction

k) Professional, Technical or Business Services

l) Real Estate or Rental and Leasing Services

m) Retail Trade

n) Transportation or Warehousing

o) Utilities

p) Wholesale Trade

q) Government, including all federal and state

r) Other (please specify) [text input]

26. What is your ***occupation?***

Multiple choice | Required | Vertical | Single-select

a) Armed forces

b) Construction and extraction (e.g. mining)

c) Farming, fishing, and forestry

d) Installation, maintenance and repair

e) Management, business and financial

f) Office and administrative support

g) Production

h) Professional and related

i) Sales and related

j) Service

k) Transportation and material moving

l) Other (please specify) [text input]

[Q27 logic: Show if Q1 (Block 2) selected choice is "Working for pay, whether on business premises or working from home"]

27. How many ***hours*** did you ***work for pay last week?***

Number | Required | Min: 0 | Max: 100 | Decimals: 1

______ hours

28. What is the ZIP/Postal code of your ***current*** residential address?

Text input | Required | Single line

Require valid zip code

Min length: 1

Max length: 10

[Q29 logic: Show if Q1 (Block 2) selected choice is "Working for pay, whether on business premises or working from home"]

29. What is the ZIP/Postal code of your ***current*** job's business premises?

    Text input | Required | Single line

    Require valid zip code

    Min length: 1

    Max length: 10

30. On the days that you commute to work, how much time (in minutes) does it typically take***?***

    Combination | Required | Answer option randomization

Time commuting to work<br>Time commuting back from work<br><br><br><br>I do not commute because I work fully remote<br>I do not commute because I do not work at the moment	**Number input**<br>_______ minutes<br>Min: 0<br>Max: 240

[Q31 logic: Show if answer option "I do not commute because I work fully remote" is chosen in Q30]

31. How long would it take to travel (one-way) from your home to your employer's location?

    Number | Required | Min: 0 | Max: 12 | Decimals: 1

    _______ hours

[Q32 logic: Show if Q23 (Block 2) selected choice is "Yes"]

32. When you work from home, how do you **spend the time you save by not commuting**? Please assign a percentage to each activity (the total should add to 100%).
    Allocation | Required | Answer option randomization | Total: 100

    a) Working on my main job

    b) Working on my second job

c) Childcare

d) Home improvement, chores, or shopping

e) Leisure indoors (e.g. reading, watching TV and movies)

f) Exercise or outdoor leisure

g) Sleep

[Q33 logic: Show if Q22 (Block 2) selected choice is none of "I am self-employed and run my own business", "I earn most of my income as an independent contractor, freelancer, or gig worker."]

33. Think about your primary employer's worksite. How many employees work there for your primary employer?

Multiple choice | Required | Vertical | Single-select

a) Fewer than 10

b) 10-24

c) 25-49

d) 50-99

e) 100-249

f) 250-499

g) 500-999

h) 1,000 or more

[Q33a logic: Show if Q22 (Block 2) selected choice is none of "I am self-employed and run my own business", "I earn most of my income as an independent contractor, freelancer, or gig worker."]

33a. Does your primary employer operate more than one worksite?

Multiple choice | Required | Vertical | Single-select

a) Yes

b) No

[Q33b logic: Show if Q33a selected choice is "Yes"]

33b. How many employees does your primary employer have across all of its worksite locations?

Multiple choice | Required | Vertical | Single-select

a) Fewer than 10

b) 10-24

c) 25-49

d) 50-99

e) 100-249

f) 250-499

g) 500-999

h) 1,000 to 4,999

i) 5,000 or more

[Q33c logic: Show if Q22 (Block 2) selected choice is none of "I am self-employed and run my own business", "I earn most of my income as an independent contractor, freelancer, or gig worker."]

33c. Where is your primary employer's global headquarters located? That is, the location where the chief executive or top manager usually works.

Multiple choice | Required | Vertical | Single-select

a) In the country where I live

b) In some other country

[Q33d logic: Show if Q22 (Block 2) selected choice is "I am self-employed and run my own business" or "I earn most of my income as an independent contractor, freelancer, or gig worker."]

33d. For your self-employment, contract, or freelance work, where are the majority of your clients located?

Multiple choice | Required | Vertical | Single-select

a) My home country

b) Abroad

[Q33e logic: Show if Q22 (Block 2) selected choice is none of "I am self-employed and run my own business", "I earn most of my income as an independent contractor, freelancer, or gig worker." AND Q33c selected choice is "In some other country"]

33e. Where is the company headquarters of your primary employer located?

Please specify the country: **[dropdown country list]**

[Q33f logic: Show if Q1 (Block 2) selected choice is any of "Working for pay, whether on business premises or working from home", "Still employed and paid, but not working"]

33f. Please estimate what share of your company's sales comes from sales abroad?

Multiple choice | Required | Vertical | Single-select

a) More than 75%

b) 50-74%

c) 25-49%

d) Less than 25%

[Q34 logic: Show if Q22 (Block 2) selected choice is "I am self-employed and run my own business"]

34. How many people does your business employ (excluding yourself)?

Q_num_employees | Number | Required | Min: 0 | Max: 1000

___ employees

35. How reliable is your internet connection at home?

Multiple choice | Required | Vertical | Single-select | Randomize whether the answer option order is a-b-c-d-e or reversed order

a) Perfect, the internet works 100% of the time (never experience outages)

b) Good, the internet works 90% of the time (rarely experience outages)

c) Moderate, the internet works 70% to 80% of the time (occasionally experience outages)

d) Poor, the internet works less than 70% of the time (often experience outages)

e) None, I have no internet connection at home

Block 3:

[Q1 logic: Show if Q2 (Block 2) yields at least 1, but less than 5 "Worked from home" days]

1. What prevents you from doing more of your tasks remotely? Please select all that apply.
Multiple choice | Required | Vertical | Multi-select
[randomize answer options, excluding the last answer option]

- Tasks that require physical presence
- Inadequate internet infrastructure
- Legal or regulatory constraints
- Company or organizational policy
- I personally prefer not to work at home

- Other (please specify): __

[Q2 logic: Show if Q2 (Block 2) yields no “Worked from home” days AND chosen answer in Q2 (Block 1) is a), b) or c)]

2. What prevents you from doing your tasks remotely? Please select all that apply.
   Multiple choice | Required | Vertical | Multi-select
   [randomize answer options, excluding the last answer option]

- Tasks that require physical presence
- Inadequate internet infrastructure
- Legal or regulatory constraints
- Company or organizational policy
- I personally prefer not to work at home
- Other (please specify): __

3. Where do you currently live?

   Multiple choice | Required | Vertical | Single-select

Please specify the type of city/town:

- City/town with less than 2,000 inhabitants
- City/town with 2,000 to 50,000 inhabitants
- City/town with 50,000 to 250,000 inhabitants
- City/town with 250,000 to 1 million inhabitants
- City/town with more than 1 million inhabitants

Please also specify the type of location:

- City center
- Suburb
- Rural area

4. Have you moved from another country, or another city or region within your country in the last ten years? Please select all that apply.
   Multiple choice | Required | Vertical | Multi-select

- Yes, from another country
- Yes, from another region or city in my country
- No, I did not move

[Q4a-Q4d logic: Show if Q4 was answered “Yes, from another country” or “Yes, from another region or city in my country”]

4a. When did you move? If you have moved multiple times in the last 10 years, please specify the year of your latest move.

Please specify the year: _________

[Q4b logic: Show If Q4 was answered "Yes, from another country":]

4b. Where did you move from?

Multiple choice | Required | Vertical | Single-select

Please specify the country: **[dropdown country list]**

[Q4c logic: Show if Q4 was answered "Yes, from another region or city in my country":]

4c. Where did you move from?

Multiple choice | Required | Vertical | Single-select

Please specify the type of city/town:

- City/town with less than 2,000 inhabitants
- City/town with 2,000 to 50,000 inhabitants
- City/town with 50,000 to 250,000 inhabitants
- City/town with 250,000 to 1 million inhabitants
- City/town with more than 1 million inhabitants

Please also specify the type of location:

- City center
- Suburb
- Rural area

4d. Why did you move? If you have moved multiple times in the last 10 years, please refer to your latest move. Please select all that apply.

[randomize answer options]

Multiple choice | Required | Vertical | Multi-select | Answer option randomization

- Remote work opportunities
- Employment opportunities
- Lower cost of living
- Family reasons
- Health or safety concerns
- Access to amenities (shops, restaurants, etc.)
- Availability of outdoor spaces (parks, trails, etc.)
- Educational opportunities
- Financial considerations

5. Do you plan to move permanently to another city within your country or to another country, or would you prefer to continue living in your current city?
   Multiple choice | Required | Vertical | Single-select
   a. Yes, to another city or region within my country
   b. Yes, to another country
   c. No, I prefer to stay in my city

[Q5a-Q5f logic: Show if Q5 was answered "Yes, to another city or region within my country" or "Yes, to another country"]

5a**.** When do you plan to move?

Multiple choice | Required | Vertical | Single-select

- Within the next six months
- Within the next 6 to 12 months
- Within the next 1 to 3 years
- I do not have a specific time frame

[Q5b logic: Show if Q5 was answered "Yes, to another region or city within my country":]

5b. Where would you like to move?

Multiple choice | Required | Vertical | Single-select

Please specify the type of city/town:

- City/town with less than 2,000 inhabitants
- City/town with 2,000 to 50,000 inhabitants
- City/town with 50,000 to 250,000 inhabitants
- City/town with 250,000 to 1 million inhabitants
- City/town with more than 1 million inhabitants

Please also specify the type of location:

- City center
- Suburb
- Rural area

[Q5c logic: Show If Q5 was answered "Yes, to another country"]

5c. Where would you like to move?

Multiple choice | Required | Vertical | Single-select

Please specify the country: **[dropdown country list]**

5d. Why are you planning to move? Please select all that apply. **[randomize answer options]**

Multiple choice | Required | Vertical | Multi-select | Answer option randomization

- Remote work opportunities
- Employment opportunities
- Lower cost of living
- Family reasons
- Health or safety concerns
- Access to amenities (shops, restaurants, etc.)
- Availability of outdoor spaces (parks, trails, etc.)
- Educational opportunities
- Financial considerations

5e. Have you done any preparation for this move?

Multiple choice | Required | Vertical | Single-select | Answer option randomization

- Yes
- No

[Q5f logic: Show If Q5e was answered “Yes”]

5f. Which of the following preparations have you done for your move? Please select all that apply.

Multiple choice | Required | Vertical | Multi-select

[randomize answer options, excluding the last answer option]

- Looked for a place to live
- Contacted friends, relatives or professional contacts in the new location
- Researched local culture and laws
- Planned for healthcare coverage
- Updated or obtained a passport/employment visa
- Looked for a job or source of income in the new location
- None of the above

6. In general, how willing or unwilling are you to take risks, using a scale from 0 to 10, where 0 means you are “completely unwilling to take risks” and 10 means you are “very willing to take risks.”

7. How willing are you to give up something that is beneficial for you today in order to benefit from that in the future?

   0 (= completely unwilling) to 10 (very willing)

8. How well does the following statement describe you as a person? I assume that people have only the best intentions.

   0 (=does not describe me at all) to 10 (describes me perfectly)

9. What year were you born? __

   Number | Required | Min: 1959 | Max: 2005
